# Dangers of the Defaults: A Tutorial on the Impact of Default Priors When Using Bayesian SEM With Small Samples

**DOI:** 10.3389/fpsyg.2020.611963

**Published:** 2020-12-11

**Authors:** Sanne C. Smid, Sonja D. Winter

**Affiliations:** ^1^Department of Methodology and Statistics, Utrecht University, Utrecht, Netherlands; ^2^Department of Psychological Sciences, University of California, Merced, Merced, CA, United States

**Keywords:** Bayesian SEM, default priors, informative priors, small sample size, Shiny app

## Abstract

When Bayesian estimation is used to analyze Structural Equation Models (SEMs), prior distributions need to be specified for all parameters in the model. Many popular software programs offer default prior distributions, which is helpful for novel users and makes Bayesian SEM accessible for a broad audience. However, when the sample size is small, those prior distributions are not always suitable and can lead to untrustworthy results. In this tutorial, we provide a non-technical discussion of the risks associated with the use of default priors in small sample contexts. We discuss how default priors can unintentionally behave as highly informative priors when samples are small. Also, we demonstrate an online educational Shiny app, in which users can explore the impact of varying prior distributions and sample sizes on model results. We discuss how the Shiny app can be used in teaching; provide a reading list with literature on how to specify suitable prior distributions; and discuss guidelines on how to recognize (mis)behaving priors. It is our hope that this tutorial helps to spread awareness of the importance of specifying suitable priors when Bayesian SEM is used with small samples.

Bayesian estimation of Structural Equation Models (SEMs) has gained popularity in the last decades (e.g., Kruschke et al., 2012; van de Schoot et al., 2017), and is more and more often used as a *solution* to problems caused by small sample sizes (e.g., McNeish, 2016a; König and van de Schoot, 2017)^1^. With small samples, frequentist estimation [such as (restricted) Maximum Likelihood or (weighted) least squares estimation] of SEMs can result in non-convergence of the model, which means that the estimator was unable to find the maximum (or minimum) for the derivative of the model parameters. Even when a model converges, simulation studies have shown that the parameter estimates may be inadmissible (e.g., Heywood cases) or inaccurate (i.e., the estimate deviates from the population value; Boomsma, 1985; Nevitt and Hancock, 2004). In contrast to frequentist methods, Bayesian methods do not rely on large sample techniques, which make Bayesian methods an appealing option when only a small sample is available. Within the Bayesian framework, prior distributions need to be specified for all parameters in the model^2^. This additional step may pose a barrier for novice users of Bayesian methods. To make Bayesian SEM accessible to a broad audience, popular software programs for analyzing Bayesian SEMs, such as M*plus* (Muthén and Muthén, (1998–2017)) and the blavaan package (Merkle and Rosseel, 2018) in R (R Core Team, 2018), offer default prior distributions. However, those default prior distributions are not suitable in all cases. When samples are small, the use of solely default priors can result in inaccurate estimates—particularly severely inaccurate variance parameters—unstable results, and a high degree of uncertainty in the posterior distributions (e.g., Gelman, 2006; McNeish, 2016a; Smid et al., 2019b). These three consequences of using default priors with small samples severely limit the inferences that can be drawn about the parameters in the model.

With small samples, the performance of Bayesian estimation highly depends on the prior distributions, whether they are software defaults or specified by the researcher (e.g., Gelman et al., 2014; Kaplan, 2014; McElreath, 2016). McNeish (2016a) discussed that small sample problems (such as non-convergence, inadmissible and inaccurate parameter estimates) cannot be fixed by only switching from a frequentist to a Bayesian estimator. Instead, he argues that if Bayesian methods are used with small samples, “prior distributions must be carefully considered” (McNeish, 2016a, p. 764). This advice is not new: Kass and Wasserman (1996) already warned against relying on default prior settings with small samples. In the quarter-century since that initial warning, Bayesian estimation is increasingly used to deal with small samples (van de Schoot et al., 2017; Smid et al., 2019b). Yet researchers remain stubbornly reliant on default priors, despite clear caution against their use (as shown by McNeish, 2016a; König and van de Schoot, 2017; van de Schoot et al., 2017).

## Goals of This Tutorial Paper

In this tutorial paper, we provide a non-technical discussion of the risks associated with the use of default priors. We discuss how default priors can unintentionally behave as highly informative priors when samples are small. Next, we demonstrate an educational online Shiny app (available on our Open Science Framework (OSF) page via https://osf.io/m6byv), in which users can examine the impact of varying prior distributions and sample size on model results. We discuss how the Shiny app can be used in teaching and provide an online reading list (available via https://osf.io/pnmde) with literature on Bayesian estimation, and particularly on how to specify suitable prior distributions. Finally, we provide guidelines on how to recognize (mis)behaving priors.

## What Is a Small Sample?

Before we continue our discussion of the potential dangers of default priors with small samples, we need to address the question: What exactly *is* a small sample? Whether a sample is small depends on the complexity of the model that is estimated. One way to express the size of a sample is to look at the ratio between the number of observations and the number of unknown parameters in the model (e.g., Lee and Song, 2004; Smid et al., 2019a). A sample could be considered very small when this ratio is 2, which means there are just two observations for each unknown parameter. As SEMs often include many unknown parameters (i.e., factor loadings, intercepts, covariances), samples that may appear relatively large are in fact very small. For example, a confirmatory factor analysis (CFA) model with three latent factors and fifteen observed items consists of 48 unknown parameters: 12 factor loadings (first factor loading fixed at 1 for identification), 15 intercepts, 15 residual variances, three factor variances, and three factor covariances. In this scenario, a sample of 100 participants would still be considered very small (ratio = 2.08). This example demonstrates that general rules of thumb about sample sizes for SEM (e.g., *n* > 100; Kline, 2015) can be misleading as they do not take into account model complexity. Furthermore, model complexity depends on more than just the number of parameters that are estimated. Other factors that play are role in model complexity are whether the model includes components such as categorical variables, latent factors, multiple groups, or latent classes. A recent review of simulation studies on SEM (Smid et al., 2019b) showed that authors of these simulation papers have widely varying definitions of a “small sample size,” ranging from extremely small (e.g., *n* = 8 assessed at three time points with one continuous variable; van de Schoot et al., 2015) to what some might consider moderately sized (e.g., *n* = 200 with 12 ordinal variables; Chen et al., 2015). Thus, assessing whether a sample is (too) small is unfortunately not as easy as checking whether a certain number of participants has been reached, and should be done on an analysis-by-analysis basis.

## Dangers of the Defaults

The risks associated with default priors when Bayesian SEM is used with small samples can be described as a combination of the following three factors.

**FIGURE 1:**
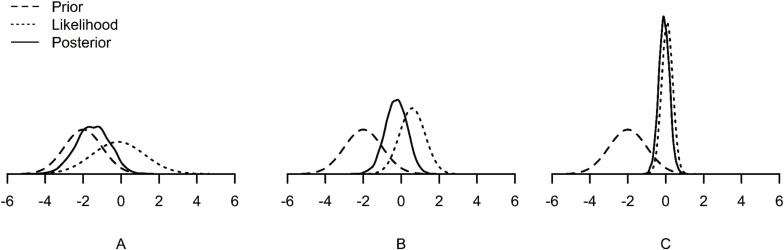
Examples of prior, likelihood and posterior distributions under small **(A)**, medium **(B)**, and large **(C)** sample sizes. The posterior distribution is dominated by the prior under the small sample size **(A)**, and dominated by the likelihood under the large sample size **(C)**.

First, when samples are small, priors have a relatively larger impact on the posterior than when samples are large. The posterior can be seen as a compromise between the prior and the likelihood. With a larger sample size, the likelihood dominates the posterior (see Figure 1C). However, with a small sample size, the likelihood has relatively less weight on the posterior. Accordingly, the prior has relatively more weight on the posterior (see Figure 1A). Therefore, it is of great importance to specify suitable prior distributions when samples are small (e.g., Gelman et al., 2014).

Second, most of the default priors have very wide distributions. For instance, the M*plus* default prior for means and regression coefficients is a Normal distribution with a mean hyperparameter of zero and a variance of 10^10^ (Muthén and Muthén, (1998–2017)). The variance hyperparameter corresponds to a standard deviation of 100.000, meaning, that 68% of the prior distribution contains values between −100.000 and 100.000, and 95% of the prior distribution contains values between −200.000 and 200.000^3^. When such default priors are specified, a wide range of parameter values can be sampled from the posterior during the Bayesian analysis. All those parameter values are therefore considered plausible, which might not always be appropriate. For instance, when measuring mathematical ability on a scale from 0 to 100, values below 0 and above 100 cannot be present in the data. Specifying a default prior with such a wide distribution on the mean of mathematical ability will put a lot of weight on values that are not reasonable (see e.g., Stan Development Team, 2017 p. 131). For small sample sizes, the combination of the relatively larger impact of the prior on the posterior *and* the wide distribution of default priors can lead to extremely incorrect parameter estimates (see e.g., Gelman, 2006; McNeish, 2016a; and the systematic literature review of Smid et al., 2019b).

The third factor that plays a role, is the *false belief* that default priors are non-informative priors which “let the data speak.” Default priors can *act* as highly informative priors, as they can heavily influence the posterior distribution and impact the conclusions of a study (see e.g., Betancourt, 2017). As explained by McNeish (2016a, p. 752): *“with small samples, the idea of non-informative priors is more myth than reality* (…).” The terminology of informative and non-informative priors can therefore be confusing (see also Bainter, 2017, p. 596). In addition, different software programs use different default priors (see Table 1). van Erp et al. (2018, p. 26) investigated the performance of multiple default priors and concluded that, especially with small samples, all investigated default priors performed very differently, and *“that there is not one default prior that performed consistently better than the other priors* (…).” The choice of software could thus unintentionally influence the results of a study (see e.g., Holtmann et al., 2016), which is problematic if one is not aware of this. Note that we are not advocating against default priors in general. Default priors can be suitable—even when samples are small—in cases where all values in the prior distribution are reasonable and can occur in the data (for example values around 100,000 or 200,000 are realistic in housing price data, see e.g., LeGower and Walsh, 2017). However, the use of default priors is problematic when researchers assume they let “the data speak” while in reality they “let the default priors speak,” meaning that the priors can heavily impact the results without one being aware of this.

**TABLE 1:** Overview of default prior distributions of main parameters for the software program Mplus and the use of Mplus, JAGS and Stan via the R package blavaan.

	M*plus* (v. 8.4) Priors on variance σ^2^	Blavaan (v. 0.3-8) Priors on precision 1/σ^2^ or standard deviation σ denoted by (SD)
Observed variable intercept	N(0, 10^10^)	N(0, 32)
Latent variable intercept, factor loading, and regression	N(0, 10^10^)	N(0, 10)
Variance covariance blocks of size 1	IG(–1, 0)	
Variance covariance blocks of size larger than 1	IW(0, –*p* –1), where *p* is the size of the matrix	
Observed and latent variable variance		G(1, 0.5)^1^
Covariance matrix		W(3, I)^2^
Correlation		B(1, 1)
Threshold	N(0, 10^10^)	N(0, 3.16)

*Default priors corresponding to Mplus version 8.4 (see Asparouhov and Muthén, 2010), and blavaan version 0.3-8 (see Merkle, 2019). Prior distributions in Mplus are placed on the variance, while the prior distributions in blavaan are by default placed on the precisions (the inverse of the variance) unless stated otherwise. Abbreviations in order of appearance: N, Normal distribution with hyperparameters mean μ and variance σ^2^; I, Identity Matrix; IG, Inverse Gamma; G, Gamma; IW, Inverse Wishart; W, Wishart; B, Beta distribution.^1^The prior for the observed and latent variable parameters is placed on the standard deviation (the square root of the variance).^2^In blavaan, three MCMC packages can be used (target = “stan,” “stanclassic” and “jags”) for the analysis. For all the MCMC packages, the same default priors are specified, with one exception: for target = “jags,” a different prior for the covariance is specified.*

In the next section, we discuss the Shiny app that we developed to demonstrate in an example the possible *informative behavior* of default priors when the sample is small.

## Shiny App: The Impact of Default Priors

**FIGURE 2:**
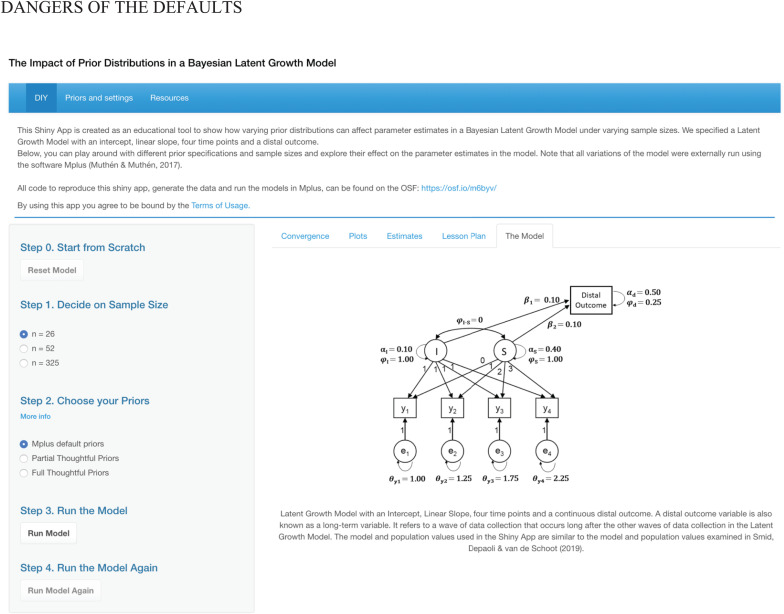
Main page of the Shiny app, where users can interactively explore the impact of prior settings and sample size in a Bayesian Latent Growth Model.

We have created a Shiny app that serves as an educational tool that can be used to learn more about the impact of default priors in Bayesian SEM. It can be found online via https://osf.io/m6byv, together with supplementary files and R code to reproduce the app. In addition, we have created a lesson plan (available for download in the app) to support the educational focus of the app. The app consists of three pages: (1) a page where users can interactively explore the impact of prior settings and sample size on a Bayesian latent growth model (see Figure 2), (2) an overview of the prior specifications used in the app, and (3) a list of further resources to learn more about various aspects of Bayesian SEM. The main, interactive, page includes a menu that walks users through selecting their sample size, prior specification settings, and running the model a first time and a second time with a doubled number of iterations (in line with the WAMBS checklist of Depaoli and van de Schoot, 2017). The models in the Shiny app were externally run using the software M*plus* (Muthén and Muthén, (1998–2017)) to enhance the user experience^4^.

The main window on the page has five tabs that can be used to (1) see what model is estimated, (2) check convergence of the model using the potential scale reduction factor (PSFR; Gelman and Rubin, 1992), examine the precision of the posterior samples with the effective sample size (ESS), (3) look at plots of the prior, likelihood, and posterior and trace plots, (4) inspect parameter estimates, (5) access the lesson plan.

### The Model, Sample Sizes, and Priors Used in the Shiny App

**FIGURE 3:**
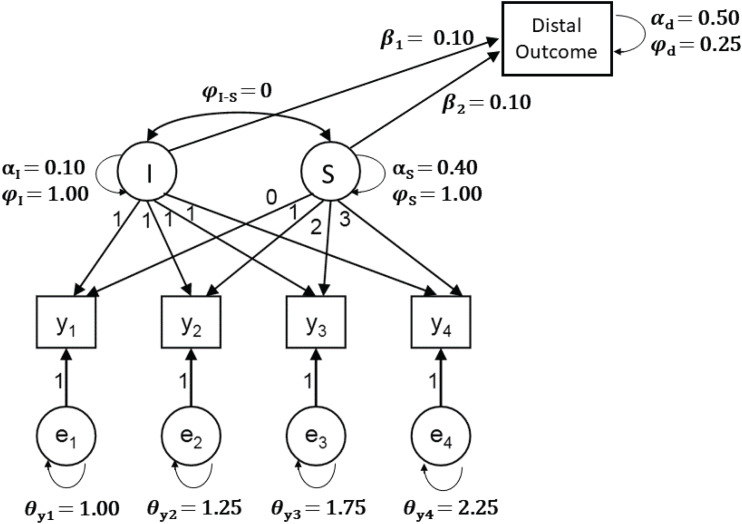
The Latent Growth Model with a distal (long-term) outcome variable that is used in the Shiny app, including population values (model and population values based on Smid et al., 2019a).

The model, sample sizes, and prior settings used in the Shiny app are based on Smid et al. (2019a). Specifically, the model is a latent growth model (LGM) with a latent intercept and linear slope, four time points, and a continuous long-term variable (i.e., distal outcome) that is predicted by the latent intercept and slope (see Figure 3). A long-term variable is a variable that is collected at a wave of assessment that occurs long after the other waves of assessment in the LGM. An example of a distal outcome is young adult levels of depression that are predicted by conduct and emotional problems at ages 4–16 (Koukounari et al., 2017). Users can select one of three sample sizes: 26, 52, 325, which represent a very small, small, and relatively large sample for the model of interest, which has 13 unknown parameters.

Three different prior specifications are included in the app: one specification using software default priors and two specifications with increasing numbers of thoughtful priors. The default priors that we selected are those specified in M*plus* (Muthén and Muthén, (1998–2017)) and are called “M*plus* default priors” in the Shiny app. The two thoughtful prior specifications, called “Partial Thoughtful Priors” and “Full Thoughtful Priors,” were taken from Smid et al. (2019b), details of which are included on the second page of the Shiny app. In short, “Partial Thoughtful Priors” includes informative priors for the mean of the intercept and slope of the LGM, the regression coefficients, and the intercept of the distal outcome. “Full Thoughtful Priors” includes informative priors on all parameters in the model, with the exception of the residual variances. These two specifications reflect scenarios where a researcher has access to prior knowledge regarding some or most of the parameters in the model.

The specific hyperparameter values of the thoughtful priors (e.g., where the center of the prior is and how narrow the prior is) in the example used in the app are somewhat arbitrary because they are based on a simulation study. Specifically, the priors are all centered around the (known) population values and the width of the priors is based on the width of the posterior distribution of the analysis done with M*plus* default priors. This approach is most closely related to a type of prior specification called data dependent prior specification (McNeish, 2016b), where an initial analysis using default priors or frequentist estimation methods provides the values for the prior hyperparameters. In applied research, data dependent priors are controversial, as the researcher technically double-dips by using their data to specify the priors that are subsequently used to analyze their data (Darnieder, 2011). To resolve this issue, researchers could split their data in half and base the prior specification for the Bayesian analysis on the results of a frequentist analysis using 50% of the total sample. As this approach would further reduce the sample size for the final analysis, this approach for specifying priors may not be feasible with small sample sizes.

The two thoughtful prior specifications included in the app are just two examples of how thoughtful priors can be included in Bayesian SEM. Other sources that can be used for specifying thoughtful priors include previous research, meta-analyses, or knowledge from experts in the field (for in-depth discussions of these topics, we refer to Zondervan-Zwijnenburg et al., 2017; Lek and van de Schoot, 2018; van de Schoot et al., 2018). Even if prior knowledge is not readily available, researchers can think about impossible and implausible values for the parameters and specify prior distributions that only contain information about the typical range of the parameters. To illustrate this idea, imagine that the distal outcome of the LGM shown in Figure 3 was measured with a questionnaire that had a range from 0 to 20. A researcher could use this information to specify a prior for the intercept of the distal outcome that makes values outside of that range highly improbable [e.g., *N*(10, 15)]. For some parameters, it may be challenging to identify prior hyperparameters that will exclude implausible values. For example, the inverse Gamma distribution is often used as a prior for the (residual) variance parameters. The parameters of this distribution, called shape and scale, are not as easily interpreted and thoughtfully specified as the mean and variance of a normal distribution. Fortunately, methods for specifying thoughtful prior hyperparameters for the inverse Gamma distribution have been suggested (e.g., Zitzmann et al., 2020). Alternatively, researchers may decide to switch to a different distribution altogether (van Erp et al., 2018). Examples include the half-Cauchy prior (Gelman, 2006; Polson and Scott, 2012) or reference priors such as Jeffrey’s prior (Tsai and Hsiao, 2008).

### Using the Shiny App as a Teacher

Since this Shiny app was explicitly developed to serve as an educational tool, we have created a worksheet and answer key that can be downloaded directly in the app itself^5^. In addition, it is possible within our app to export all plots and tables created. These can be used in answering the questions on the worksheet. By making students aware of the impact of relying on default settings when samples are small, we hope to teach students about the importance of specifying suitable prior distributions and to contribute to the responsible use of Bayesian SEM.

## Guidelines: How to Recognize a (Mis)Behaving Prior?

To formulate suitable prior distributions *and* to check afterward whether the priors are “behaving,” information is needed about the reasonable range of values for the parameters in the model. This information can be based on previous studies, the scale or questionnaire that is used, or expert knowledge from the field. In our reading list (available via https://osf.io/pnmde), we provide an overview of relevant literature on how to specify suitable priors based on multiple sources of information. Below, we discuss four ways to identify a (mis)behaving prior after conducting a Bayesian analysis (see also Table 2), by inspecting for all parameters the (a) effective sample size, (b) trace plots, (c) prior-likelihood-posterior distributions, and (d) the posterior standard deviation and 95% highest posterior density.

**TABLE 2:** Possible signs of “misbehaving” priors.

**Effective sample size**
-Low effective sample size (i.e., < 1,000) can be a first indication that the priors are problematic
**Trace plots**
- Spikes: shape of alien communication captured in a sci-fi movie instead of a fat caterpillar - Highly improbable values for the parameter on the y-axis based on information about the reasonable range of values about parameters - Chains that are not overlapping
**Prior-likelihood-posterior comparison**
- Substantial deviation between prior, likelihood and/or posterior: e.g., a posterior that is much narrower or wider than the prior and likelihood, while taking into account the amount of information in the prior (i.e., level of informativeness of the prior) and in the likelihood (i.e., sample size)
**Posterior SD and 95% HPD**
- Much smaller or larger posterior SD or 95% HPD than expected based on the amount of information in the prior (i.e., level of informativeness of the prior) and in the likelihood (i.e., sample size)

### Effective Sample Size

Inspecting the effective sample size (ESS) of each parameter in the model is a good first step in the search for misbehaving priors. The ESS represents the number of independent samples that have the same precision as the total number of samples in the posterior chains (Geyer, 1992). The ESS is closely related to the concept of autocorrelation, where current draws from the posterior distribution are dependent on previous draws from the posterior distribution. Autocorrelation is undesirable as it increases the uncertainty in posterior estimates. If autocorrelation within the chains is low, then the ESS approaches the total number of samples in the posterior chains, and the posterior distribution will be more precise and more likely to approximate the parameter estimate well (Zitzmann and Hecht, 2019). If autocorrelation within the chains is high, a larger number of samples will be necessary to reach an adequate ESS. A low ESS can be the first indicator that there might be a misbehaving prior. Multiple recommendations have been made about how to assess whether the ESS is *too* low: Zitzmann and Hecht (2019) recommend that ESSs should ideally be over 1,000 to ensure that there is enough precision in the chain. It is also possible to compute a lower bound for the number of effective samples required using a desired level of precision and the credible interval level of interest (Vats et al., 2019; Flegal et al., 2020). Finally, it can also be helpful to look at the ratio of the ESS to the total number of samples, where a ratio < 0.1 indicates that there are high levels of autocorrelation in the chains (although this does not necessarily indicate that the posterior distribution is not precise; Gabry et al., 2019). A low ESS can serve as the first clue that something might be wrong, but even if all ESSs appear acceptable, plots and posterior estimates should be inspected to further confirm if priors are behaving.

### Trace Plots

**FIGURE 4:**
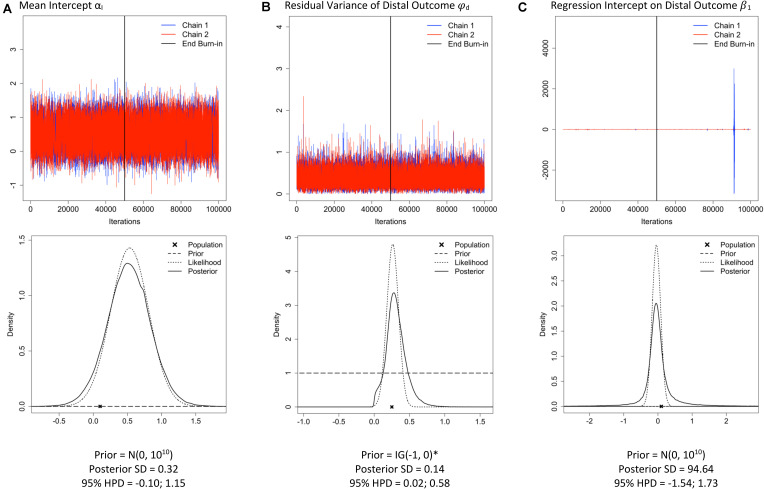
Traceplots; prior, likelihood, posterior plots; posterior standard deviation (SD) and 95% highest posterior density interval (HPD) for three parameters: mean intercept **(A)**, residual variance of the distal outcome **(B)** and the regression effect of the slope on the distal outcome **(C)** under sample size *n* = *26* and M*plus* default priors (examples retrieved from the Shiny app). *The M*plus* default prior for residual variance parameters is IG(−1, 0), which is improper (i.e., does not integrate to 1) and has a constant density of 1 on the interval (−∞, ∞) (Asparouhov and Muthén, 2010).

Three characteristics of a trace plot can indicate a misbehaving prior. First, the shape of the trace plot: If the multiple chains are well-behaved, the chains should resemble the hungry caterpillar after 6 days of eating (see Figure 4A). A misbehaving prior can result in trace plots that exhibit spikes, closely resembling alien communication captured in a sci-fi movie (Figure 4C). Second, do the values that are covered by the posterior make sense for this parameter, or is the *y*-axis stretched to cover unrealistic values? Even when subtle spikes are present (Figure 4B), the *y*-axis range could show that the chains are drawing improbable values from the posterior distribution and should be given extra attention. Third, a lack of overlap of the chains can indicate a misbehaving prior. When the chains do not overlap, it indicates that they are sampling from different parts of the posterior distribution and are not converging toward the same location.

### Prior-Likelihood-Posterior Comparison

One important aspect of our Shiny app is that the prior, likelihood, and posterior distributions are visualized to make comparisons across different priors and sample size settings easy^6^. When there is a substantial deviation between the prior, likelihood and posterior distributions, results should be interpreted with caution, especially when the sample size is small. Researchers should decide how much impact of the prior and likelihood on the posterior is desirable. Is it preferable that the posterior is a compromise between the prior and likelihood, or that the posterior is dominated by one of two? For instance, when the likelihood and the prior deviate a lot, one might not want to trust the posterior results^7^. In case of small samples, the results might especially be driven by the prior distributions. This is only desirable when researchers trust the specified prior distributions, not when they are defaults of the software program. Figure 4 shows the prior-likelihood-posterior comparison for three parameters. Although the prior distributions (dashed lines) look completely flat, default prior distributions were used for all parameters. In Figure 4A, the posterior (solid line) closely follows the likelihood distribution (dotted line), which is desirable here because the default prior (dashed line) is specified and we do not want it to impact the posterior much. In Figures 4B,C, the posteriors seem to have tails that are too fat (kurtotic) compared to the likelihood distribution and the flat default priors, and results should therefore be inspected further.

### Posterior SD and 95% HPD

The posterior standard deviation (SD) and 95% credible (or highest posterior density; HPD) interval can be inspected to assess whether the estimates are unusually certain or uncertain. Uncertainty is demonstrated by a large posterior SD and a wide 95% HPD.

Available information about reasonable values for the parameters as well as the amount of information in the prior and likelihood should be used to assess whether the level of (un)certainty of the posterior is reasonable. For instance, in Figure 4C, a posterior SD of 94.64 is reported, which is a much higher value than would be expected for a regression estimate and implies that some very extreme values were likely sampled from the posterior. This level of uncertainty is also reflected by the extreme spikes in the trace plot and the kurtotic posterior distribution. The parameters depicted in Figure 4 illustrate that the combination of a non-informative prior and a small sample size does not always lead to problems across all parameters in a model. It is important to note that even if it appears that the priors of the main parameter(s) of interest are behaving well, a misbehaving prior that is located elsewhere in the model may lead to inaccuracies in the posterior estimates of the main parameters. For example, in a multilevel SEM with a between-level covariate effect, the between-level variance estimate may not be of substantive interest. However, a supposedly non-informative prior [IG(0.001, 0.001)] for the between-level variance parameter can turn into a misbehaving prior when the amount of variance located at the between-level is large (Depaoli and Clifton, 2015). In a simulation study, Depaoli and Clifton (2015) showed that this misbehaving prior resulted in a biased posterior estimate of the between-level covariate effect. A researcher who only inspected the trace plot for the between-level covariate effect may not have realized that their results were negatively affected by a prior placed on between-level variance parameter. For that reason, it is critical to always examine all parameters in the SEM.

### What to Do If You Suspect a Misbehaving Prior?

When one of the trace plots, prior-likelihood-posterior distribution plots, posterior SDs or 95% HPDs show signs of a misbehaving prior, results should not be trusted, and researchers should proceed with caution. Unfortunately, we cannot provide rules of thumb for when these indicators of misbehavior become problematic. It depends on the specified prior, the data, the parameter, the model of interest, and the personal judgment of the researcher. A sensitivity analysis can help assess the impact of the specified prior distributions on the posterior (see Depaoli and van de Schoot, 2017; van Erp et al., 2018). Again, it is up to the researcher to decide whether a certain amount of impact of the prior is desirable or not. Therefore, Bayesian SEM should only be used with small samples when researchers are able and willing to make these types of decisions.

### Reporting of Bayesian SEM

Although a rich body of literature exists on good practice of how to perform *and* what to report for a Bayesian analysis (see e.g., Kruschke, 2015, pp. 721–725; Depaoli and van de Schoot, 2017), we want to stress the importance of transparency and reporting every decision. We advise to always provide an (online) appendix in which is explained in detail which priors are specified and why these specific priors are chosen. For more literature and examples on reporting Bayesian SEM, we refer to our reading list on https://osf.io/pnmde.

## An Illustration: The Impact of Default Priors

**FIGURE 5:**
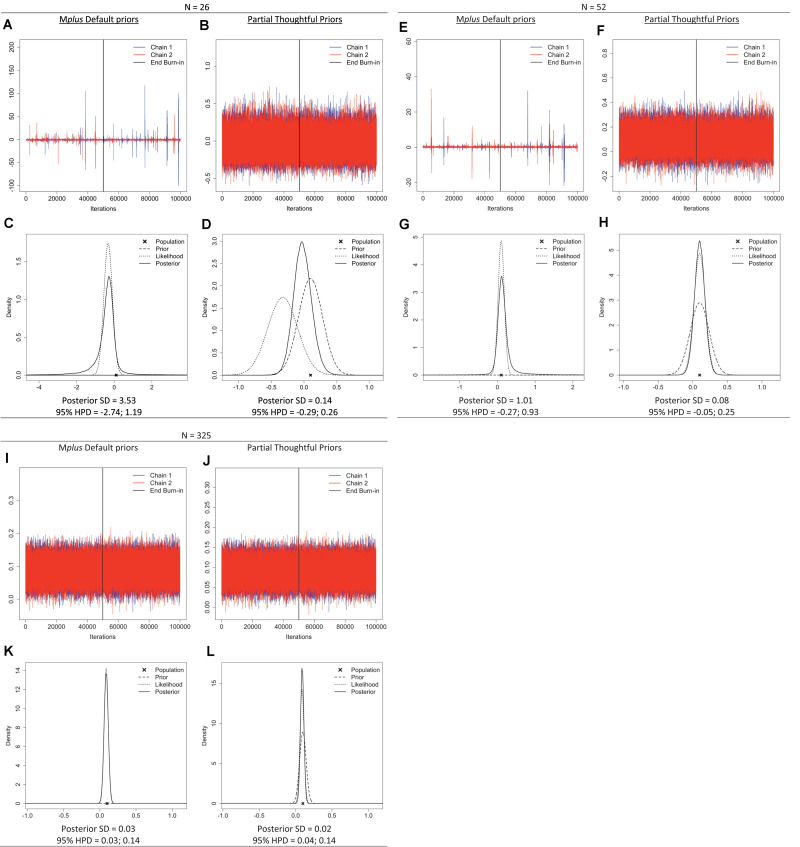
Trace plots; prior, likelihood, posterior plots; posterior standard deviation (SD) and 95% highest posterior density intervals (HPD) for regression coefficient β*_2_* under sample sizes *n* = *26, 52, 325* when M*plus* default priors and partial thoughtful priors are specified. **(A,B,E,F,I,J)** Trace plot. **(C,D,G,H,K,L)** Prior, Likelihood, Posterior Plot.

To illustrate the impact of prior settings and sample size—and the informative behavior of default priors with a small sample size—we retrieved the trace plots, prior-likelihood-posterior plots, and posterior SDs from the Shiny app for a single parameter: the regression effect of the distal outcome regressed on the linear slope (β_2_ in Figure 3). The plots (Figure 5) show signs of a misbehaving prior when samples are small (*n* = 26, or 52 for this model) when default priors are used. Specifically, the trace plots exhibit spikes that reach highly improbable values for the regression coefficient, the plots have a stretched *y*-axis, and show chains that are not overlapping. Moreover, the prior-likelihood-posterior plots for the two small sample sizes show that the posterior distribution (solid line) is wider than the likelihood estimate (dotted line). Overall, the plots displayed in Figure 5 show that default priors, which are assumed to be non-informative, can impact the results when samples are small. Options for improving model estimation include increasing the sample size or specifying suitable priors for the parameters.

## Summary

In this tutorial paper, we discussed the risks associated with default priors in Bayesian SEM when samples are small. We described the *dangers of the defaults* as a combination of three factors: (a) the relatively larger impact of the prior on the posterior when samples are small, (b) the wide distribution of default priors that often contain unrealistic values, and (c) the *false belief* that default priors are non-informative priors. We demonstrated an interactive Shiny app, in which users can investigate the impact of priors and sample size on model results. The Shiny app can also be used to teach students about responsible use of Bayesian SEM with small samples. In this paper, we showed that default priors can *act* as highly informative priors when samples are small. We provided an overview of relevant literature (available via https://osf.io/pnmde) on how to specify *suitable priors* based on multiple sources of information. We discussed how to recognize a misbehaving prior by inspecting (a) the effective sample sizes, (b) trace plots, (c) the comparison of prior-likelihood-posterior distributions, and (d) posterior standard deviation and 95% highest posterior densities.

It is important to note that we are not arguing that researchers are solely responsible for breaking away from their reliance on default priors. There are several strategies that could be employed to help researchers improve their decisions regarding prior specification. A simple way in which the use of Bayesian methods can be improved is by making available educational tools, such as the App introduced in this paper, to a broad audience of researchers. More generally, software developers could implement notifications that nudge users to check the impact of their prior distributions through techniques proposed in the current paper (e.g., flag low ESSs and suggest inspection of trace plots). Another opportunity to intervene and improve occurs during the peer-review process. Reviewers should closely examine the decisions authors have made regarding their prior specification and intervene if the decisions made by the authors were inappropriate. In such a case, a reviewer can advise that major revisions are in order to ensure that Bayesian methods were applied appropriately.

Bayesian SEM should only be used with small samples when information is available about the reasonable range of values for all parameters in the model. This information is necessary to formulate suitable prior distributions *and* to check afterward whether the priors are “behaving.” It is our hope that this tutorial paper helps spread awareness that the use of Bayesian estimation is not a *quick solution* to small sample problems in SEM, and that we encourage researchers to specify suitable prior distributions *and* carefully check the results when using Bayesian SEM with small samples.

## Author Contributions

SS designed the tutorial manuscript and shiny app, and further developed the idea of the shiny app with SW. SW worked out the code for the shiny app with input and feedback from SS. SS took the lead in writing the manuscript. SW wrote the “Shiny App” section and provided feedback on the manuscript. Both authors contributed to the article and approved the submitted version.

## Conflict of Interest

The authors declare that the research was conducted in the absence of any commercial or financial relationships that could be construed as a potential conflict of interest.

## Footnotes

1 There are many other reasons why researchers use Bayesian SEM, such as the ability to estimate models that are not identified in the frequentist framework or to resolve issues with missing data, non-linearity, and non-normality (see e.g., Wagenmakers et al., 2008; Kaplan, 2014, pp. 287–290; van de Schoot et al., 2017). However, the focus of this paper is the use of Bayesian estimation to *deal* with small samples.

2 Prior distributions represent information about the parameters and can be based on previous studies or the beliefs of experts in the field. The prior distributions are then updated by the likelihood (observed data depended on the model). By using methods such as Markov chain Monte Carlo (MCMC), the posterior distribution is simulated, which is a combination of the prior and likelihood. For references with an elaborate introduction into Bayesian estimation, we refer to our reading list (https://osf.io/pnmde).

3 Hyperparameters are the parameters of prior distributions, such as the mean and variance of the Normal distribution, and the alpha and beta in inverse gamma.

4 This popular, user-friendly software program for estimating Bayesian SEM has made it extremely easy to be a naive user of Bayesian statistics (one only needs to include the line “Estimator = Bayes;” in the input file).

5 The worksheet can be found on the main page under the fifth tab (“Lesson Plan”).

6 For details on how we visualized priors, likelihood and posterior distributions, we refer to the OSF (https://osf.io/m6byv).

7 For readers interested in the impact of so-called prior-data conflict, we refer to simulation studies by Depaoli (2014); Holtmann et al. (2016), and Smid et al. (2019a).
